# A Robust Multi-Sensor Data Fusion Clustering Algorithm Based on Density Peaks

**DOI:** 10.3390/s20010238

**Published:** 2019-12-31

**Authors:** Jiande Fan, Weixin Xie, Haocui Du

**Affiliations:** Automatic Target Recognition (ATR) Key Laboratory, Shenzhen University, Shenzhen 518060, China; jdfan@szu.edu.cn (J.F.); hcdu@szu.edu.cn (H.D.)

**Keywords:** clustering, data fusion, target detection

## Abstract

In this paper, a novel multi-sensor clustering algorithm, based on the density peaks clustering (DPC) algorithm, is proposed to address the multi-sensor data fusion (MSDF) problem. The MSDF problem is raised in the multi-sensor target detection (MSTD) context and corresponds to clustering observations of multiple sensors, without prior information on clutter. During the clustering process, the data points from the same sensor cannot be grouped into the same cluster, which is called the cannot link (CL) constraint; the size of each cluster should be within a certain range; and overlapping clusters (if any) must be divided into multiple clusters to satisfy the CL constraint. The simulation results confirm the validity and reliability of the proposed algorithm.

## 1. Introduction

As a powerful tool, clustering analysis is usually used in machine learning [1], image analysis [2], information retrieval [3] and data mining [4] to eliminate noise data-points and find hidden groups or patterns in a dataset. Due to the diversity/variability of the dataset to be processed, many clustering algorithms, such as density-based clustering [5,6], hierarchical clustering [7], and *k*-means clustering [8], have been developed to solve specific problems. It can be seen that, although there are many clustering algorithms, none of them can be applied in all cases.

Clustering is often taken as an unsupervised learning technique in many pre-processing processes, as no information is provided. Nevertheless, for many of the problems, including the MSDF clustering problem, to be solved in this paper, an amount of prior information can be obtained through additional data features [9,10], which can be employed to obtain better clustering results, namely, semi-supervised clustering.

Constraining the dataset during the clustering process to obtain specific clustering results is a hot issue in clustering research. In constrained clustering, “must-link” constraints (ML) and “cannot-link” constraints (CL) are two basic rules. An ML constraint is used to specify that the two instances should be associated with the same cluster, whereas a CL constraint is used to specify that the two instances should assigned to different clusters, allowing users to specify constraint rules to obtain the desired clustering results. Typical constrained clustering algorithms include the constrained *k*-means [11], pairwise constrained *k*-means [12], complete link [13], constrained hierarchical clustering algorithms [14].

In clustering research, the number of clusters and cluster center initialization have a great impact on the clustering convergence speed and clustering result. The research on the number of clusters mainly focuses on running the clustering algorithm multiple times, with different values of *k*, and the estimated *k* is chosen based on a specific criterion, such as the Bayesian information criterion [15], rate distortion theory [16], Akaike information criterion [17], etc. The research on cluster center initialization mainly focuses on how to maximize the distance of the initial cluster center through statistical information, such as the *k*-means++ [18]. 

In this paper, we address the problem of MSDF, which is involved in multi-sensor multi-target tracking [19], using the density peaks clustering (DPC) algorithm [5]. The DPC algorithm was published in the journal, Science, in 2014. The core idea of DPC is that cluster centers are characterized by a higher density and a relatively longer distance. The outstanding performance of DPC has attracted many scholars’ attention, and many variants based on DPC have been proposed to address various clustering problems, such as BDDPC [20], and DPC-KNN [21]. In this paper, the original DPC algorithm is shown to be able to handle the type of dataset that contains observations of targets that obey a zero-Gaussian distribution well. Therefore, we use the original DPC algorithm to solve the MSDF problem. The purpose of clustering is to divide the observations of targets into multiple clusters; overlapping clusters (if any) must be divided into multiple sub-clusters to satisfy the CL constraint; the data points in each cluster correspond to the observations of targets; and the cluster center is the estimated target position.

In the past, the MSDF problem was solved using a model-based method [22,23], but in recent years, many scholars have begun to solve the MSDF problem using a clustering-based method. Tiancheng Li has produced a lot of groundbreaking work [24,25,26,27] on this issue. He not only solves the MSDF problem using density-based clustering, but also uses multi-sensor clustering to improve the performance of a model-based filter [28]. Tianxian Zhang uses the clustering method to solve the MSTD problem with a distributed radar network [29]. This paper made several improvements on the basis of Li’s work: (1) A more accurate and robust clustering algorithm, based on DPC, is proposed; and (2) the proposed algorithm can accurately filter out clutter, and the performance of the algorithm does not vary with the change of the detection probability of the sensors, which has great advantages, given the low detection probability of sensors.

The rest of the paper is organized as follows. The problem model is discussed in Section 2. Section 3 presents details of the proposed clustering method. Section 4 discusses the experimental simulation results, which are summarized in Section 5.

## 2. Multi-Sensor Data Fusion

### 2.1. Multi-Sensor Data Fusion

The MSDF problem involves estimating the state of the unknown number and unknown motion mode targets in the presence of noise data, which has a wide application space in the remote sensing image fusion, oceanography and military fields. The general MSDF problem can be modeled by the following assumptions:

**Assumption** **1.** *Each target evolves and generates observations/measurements independently from the others.*

**Assumption** **2.** *The observations of targets obey a zero-Gaussian distribution. Both the noise and observations constitute the measurement dataset of each sensor.*

**Assumption** **3.** *One target can generate no more than one measurement in each scan.*

**Assumption** **4.** *The distribution density of the clutter is significantly lower than the density of the observations of targets.*

**Figure 1:**
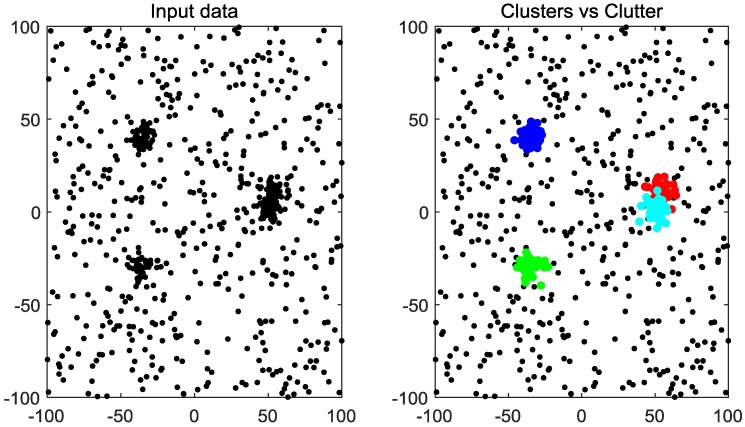
Multi-sensor i.i.d data points.

The goal of the MSDF is to distinguish the observations of each target from those of others using a clustering method, as shown in Figure 1.

### 2.2. Problem Formulation

The above MSDF problem can be formulated as a CL-constrained clustering problem. Considering a dataset Z, which consists of observations from multiple sensors, $z_{i}$ is included in dataset Z.

(1) $$z_{i}\in P,i=1,\cdots ,N$$

where parameters N and P are the number of data points and the parameter space, respectively. In this paper, we define $z_{i}$ as a point in a two-dimensional Cartesian coordinate system.

The dataset Z can be written in the form of a union of multi-sensor observations. We define the sth sensor as $S_{s}=\{z_{1}^{s},z_{2}^{s},\cdots ,z_{m_{s}}^{s}\}$, where $m_{s}$ is the number of data-points, and all the data-points in Z can be written as:

(2) $$Z:=\{S_{1},S_{2},\cdots ,S_{n}\}=\{z_{1}^{1},z_{2}^{1},\cdots ,z_{m_{1}}^{1},z_{1}^{2},z_{2}^{2},\cdots ,z_{m_{2}}^{2},\cdots ,z_{1}^{n},z_{2}^{n},\cdots ,z_{m_{n}}^{n}\}$$

where n is the number of sensors. The MSDF problem requires that the dataset Z be divided into *k* clusters, namely, $C_{1},C_{2},\cdots ,C_{k}$, and the CL constraint requires that:

(3) $$c\neq (z_{i}^{s},z_{j}^{s}),\forall i,j\in \{1,2,\cdots ,m_{s}\},s\in \{1,2,\cdots ,n\}$$

where $c\neq (z_{i}^{s},z_{j}^{s})$ means $z_{i}^{s},z_{j}^{s}$ cannot be within the same cluster.

We define the set of noisy data points in dataset Z as $C_{0}$ and the observations of targets as $C_{T}$. The dataset Z can be defined as:

(4) $$Z=C_{0}\cup C_{1}\cup C_{2}\cup \ldots \cup C_{k}=C_{0}\cup C_{T},\;k\in T$$

At the same time, each cluster cannot have any intersection with the rest of the subsets.

(5) $$C_{i}\cap C_{j}=\Phi ,\forall i,j\in \{1,2,\cdots k,0\}$$

As mentioned above, the MSDF clustering problem can be described as: A dataset Z is divided into k clusters, the size of each cluster must satisfy the CL constraint (3), and each cluster cannot have any intersection with the others (5). 

### 2.3. CL Constraint and the Size of Clusters

The CL constraint (3) limits the size of each cluster, which must be smaller or equal to the number of sensors n.

(6) $$\left|C_{i}\right|≲n,\forall i\in \{1,2,\cdots k\}$$

where $\left|C_{i}\right|$ means the number of data points in cluster $C_{i}$, ≲ means smaller than or equal to. Denoting the detection probability of the sensor s on target i as $p_{D}^{s}(i)\leq 1$, to simplify the calculation, we simplify $p_{D}^{s}(i)$ as a constant $p_{D}$, then the size of a cluster can be calculated as:

(7) $$E[\left|C_{i}\right|]=\sum_{s=1}^{n_{i}}p_{D}\leq n_{i}$$

Given $p_{D}$ and the number of sensors n, $E[\left|C_{i}\right|]$ can be considered as a constant:

(8) $$E[\left|C_{i}\right|]=r$$

The number of sub-clusters (targets) in each cluster $C_{i}$ is:

(9) $$k_{i}\approx \left[\frac{\left|C_{i}\right|}{r}\right]$$

## 3. Multi-Sensor Data Clustering Algorithm

### 3.1. Density Peaks Clustering Algorithm

In this paper, we use the DPC to calculate the local density. For each data point, we compute two quantities: its local density $\rho_{i}$ and distance $\delta_{i}$ from points of higher density. Both these quantities depend only on the distances $d_{ij}$ between data points [5]. The local density $\rho_{i}$ is defined as:

(10) $$\rho_{i}=\sum_{j}\chi (d_{ij}-d_{c})$$

where $d_{ij}$ means the distances between data points. χ(x)=1 if x<0 and χ(x)= 0; otherwise, $d_{c}$ is a cutoff distance. $\rho_{i}$ is equal to the number of data points within the cutoff distance to point i. The larger the $\rho_{i}$, the higher density of data point i, and the more likely are the observations of targets.

$\delta_{i}$ is measured by computing the minimum distance between point i and the other points with a higher density:

(11) $$\delta_{i}=\underset{j:\rho_{j}>\rho_{i}}{\min}(d_{ij})$$

**Figure 2:**
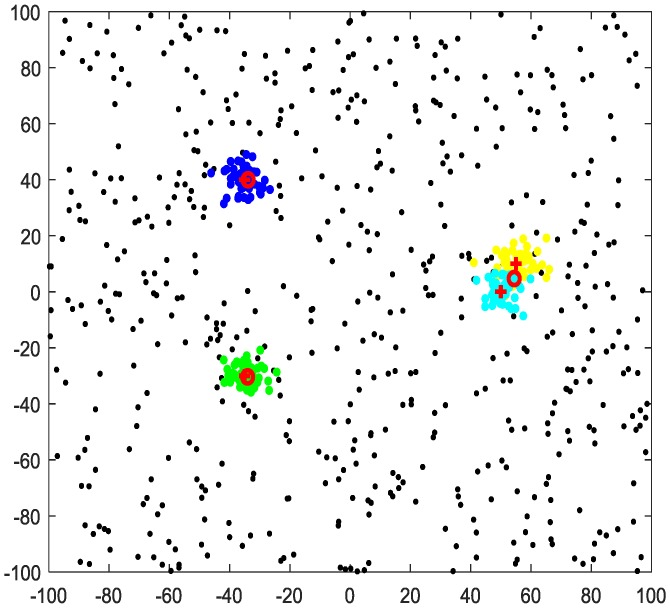
Clustering results of the density peak clustering algorithm for data from 50 i.i.d sensors.

The original DPC algorithm defines the data points of $\rho_{i}\geq 0.8\times r$ and $\delta_{i}>2d_{c}$ as cluster centers. Figure 2 shows the clustering results of the DPC algorithm of 50 i.i.d sensors. Clusters of different colors represent observations of different targets. The red “+” represents the true position of the targets, and the red “o” represents the clustering results. It can be seen, from Figure 2, that for non-overlapping clusters, the real position and estimated position of the targets are very close; for overlapping clusters, the clustering result has a large deviation from the target real position, and the target number is incorrect. In subsequent calculations, we need to re-cluster the overlapping clusters to obtain correct estimates.

From Figure 2, we can draw a conclusion: the cutoff distance in the clusters of $k_{i}\geq 2$ (overlapping clusters) is larger than that in the clusters of $k_{i}=1$ (non-overlapping clusters). We define the cutoff distance in the non-overlapping clusters as $d_{c}$, and the cutoff distance in overlapping clusters is $d_{c}’=md_{c}$,m∈[1.1,1.5]. Assuming the cluster center of $C_{i}$ is data point i,i∈{1,2,⋯,k}, the number of data points closer than i is equal to the size of the cluster $C_{i}$, that is, $\rho_{i}\approx |C_{i}|$. The data points of cluster $C_{i}$ can be defined as:

(12) $$C_{i}=\sum_{j\in \{i|d_{ij}<d_{c}\}}z_{j}$$

The number of targets in cluster $C_{i}$ can be defined as:

(13) $$k_{i}\approx \left[\frac{\rho_{i}}{r}\right]$$

The difference between Equations (9) and (13) is that Equation (13) can determine whether cluster $C_{i}$ is an overlapping cluster using the $\rho_{i}$ of cluster center i. The calculation of $k_{i}$ is also an important step in the subsequent re-clustering process.

### 3.2. Target Observations Set and Target Number

The multi-source n-points algorithm searches for the number of data points within the cutoff distance of data point i to determine whether the union of point i and the data points within the cutoff distance is a cluster formed by observations of targets. The position of the data point i and detection probability $p_{D}$ have a greater impact on the effect of the multi-source n-points algorithm. How to quickly and efficiently filter out noise and obtain the target observations is the key to designing MSDF clustering algorithms. Using the DPC algorithm, we find that data point i in $C_{T}$ has a prior rule: $\rho_{i}$ must be larger than a threshold. The data points in $C_{T}$ can be defined as:

(14) $$C_{T}=\sum_{j\in \{i|\rho_{i}\geq l\times n\}}z_{j}$$

where l=0.4 is a reference and can be chosen roughly between 0.3~0.45, $\rho_{i}\geq l\times n$ means that the number of data points closer in data points i must be larger than or equal to l×n, and the data point i in $\rho_{i}\geq l\times n$ is considered to be the target observations.

**Figure 3:**
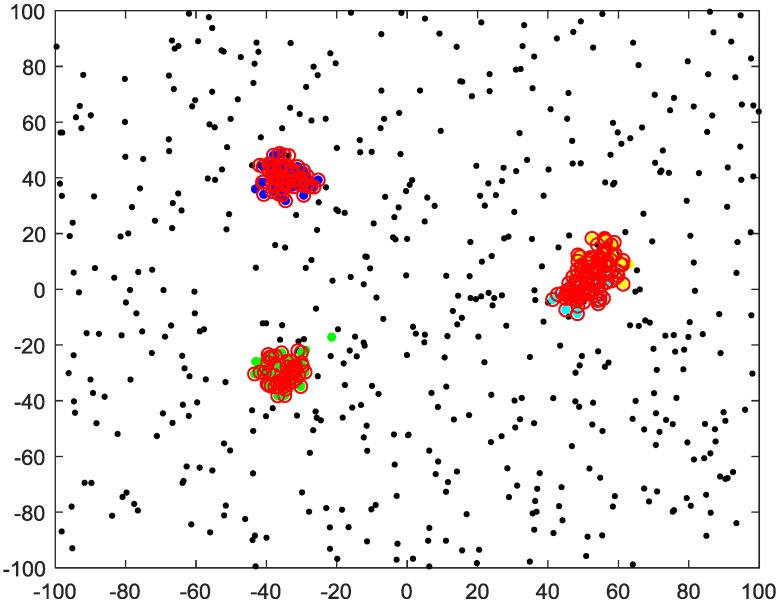
Data points of $\rho_{i}\geq l\times n$.

Based on the same dataset shown in Figure 2, the data points in $C_{T}$ are circled with a red “o” in Figure 3. As shown in Figure 3, the observations of targets (color data points) are almost circled with a red “o”, and only few data points are not circled. Considering the impact that noisy data points may have in clusters of $C_{T}$, Equation (14) is still very reliable.

The CL constraint requires that $C_{T}$ be divided into multiple clusters of roughly the same size, and the number of clusters/targets in dataset Z is:

(15) $$\sum k_{i}=\left[\frac{\left|C_{T}\right|}{r}\right]$$

The number of targets in cluster $C_{i}$ can be calculated using the $\rho_{i}$ of the cluster center i through Equation (9), while the total number of targets in dataset Z can be calculated through Equation (15).

Given the number of clusters (targets) $\sum k_{i}$ and dataset $C_{T}$, the preferred choice is to use the *k*-means algorithm for clustering, as this saves the computing resources of $\delta_{i}$; however, the *k*-means algorithm has difficulty handle cases where the local density differs greatly between clusters. During the experiment, we found that if the size of the clusters is roughly equal (no overlapping clusters), the *k*-means algorithm can obtain correct clustering results. Conversely, if there is at least one overlapping cluster contained in the dataset, the clustering result obtained by the *k*-means algorithm does not satisfy the CL constraint. In order to correctly cluster $C_{T}$ using the *k*-means algorithm, we must first determine whether there are overlapping clusters in dataset Z.

The key to determining whether there are overlapping clusters in dataset Z is to compare max(ρ) and 1.1×r. If max(ρ)<1.1×r, there are no overlapping clusters in dataset Z, and the number of targets in each cluster is 1, that is, $k_{i}=1,i\in \{1,2,\cdots ,k\}$; otherwise, at least one overlapping cluster is contained in dataset Z. The reason for why we choose 1.1×r, instead of the number of sensors n, is that the noisy data points may otherwise fall into cluster $C_{i}$.

(16) $$\left\{ \begin{array}{l} \max(\rho )<1.1\times r,k_{i}=1,i\in \{1,2,\cdots ,k\}\\ \max(\rho )\geq 1.1\times r,k_{i}\geq 2,i\in \{1,2,\cdots ,k\} \end{array}\right.$$

### 3.3. Proposed Clustering Method

The original DPC algorithm needs to calculate $\rho_{i}$ and $\delta_{i}$ to find the cluster centers, which involves a computational burden that is too great in the case of no overlapping clusters in dataset Z, in this case, we can obtain the correct clustering results using the *k*-means clustering algorithm to cluster dataset $C_{T}$ (a total of $\sum k_{i}$ targets), and dataset $C_{T}$ can be obtained by a threshold rule.

While the DPC algorithm cannot correctly cluster overlapping clusters, and we have a fast and more efficient solution for the non-overlapping clusters. For the above reasons, we divide the dataset Z into two cases for processing: (1) Non-overlapping clusters in dataset Z (Algorithm 1); and (2) at least one overlapping cluster in dataset Z (Algorithm 2). The main difference between Algorithm 1 and Algorithm 2 is that Algorithm 2 requires an additional calculation of parameter $\delta_{i}$ and re-clustering of the cluster centers of overlapping clusters.

The proposed clustering Algorithm 1 includes 3 steps: (1) Calculate the $\rho_{i}$ for each data point and determine whether there is an overlapping cluster in dataset Z according to (16); (2) filter out clutter and obtain dataset $C_{T}$ and $\sum k_{i}$ for *k*-means clustering; and (3) revisit each cluster to make sure each cluster satisfies the CL constraint.
**Algorithm 1 Clustering without any overlapping cluster in dataset**Z**Input:** dataset Z. **Output:** cluster $C_{i}$ and its cluster center $z_{i}$, i∈{1,2,⋯,k}.**1.1:** Calculate $\rho_{i}$ according to (10) and determine whether there is any overlapping cluster in dataset Z according to (16). If there is no overlapping cluster, go to step 1.2; otherwise, see Algorithm 2.**1.2:** Calculate $C_{T}$ and $\sum k_{i}$ according to (14) and (15), then cluster $C_{T}$ using the *k*-means algorithm.**1.3:** Revisit each cluster $C_{i}$ to make sure that the CL constraint was satisfied, then calculate the cluster center $z_{i}$ of each cluster.


**Algorithm 2 Clustering with at least one overlapping cluster in dataset**
Z
**Input:** dataset Z. **Output:** cluster $C_{i}$ and its cluster center $z_{i}$, i∈{1,2,⋯,k}.**2.1:** Calculate $\delta_{i}$ according to (11), and we can obtain estimated cluster centers ${\overset{⌢}{z}}_{i}$, i∈{1,2,⋯,k} using the DPC algorithm.**2.2:** According to (16), for cluster centers ${\overset{⌢}{z}}_{i}$ that are $\max(\rho_{i})<1.1\times r$, the cluster center is ${\overset{⌢}{z}}_{i}$; for cluster centers ${\overset{⌢}{z}}_{i}$ that are $\max(\rho_{i})\geq 1.1\times r$, calculate $C_{i}$ and $k_{i}$ according to (12) and (13), then cluster $C_{i}$ with the *k*-means algorithm ($k_{i}$ clusters).**2.3:** Repeat step 2.2, until all the overlapping clusters are all divided into sub-clusters.**2.4:** Revisit each cluster $C_{i}$ to make sure that the CL constraint was satisfied, then calculate the cluster center $z_{i}$ of each cluster.

**Remark** **1.** *The cutoff distance is the key to the proposed and the existing MSDF clustering algorithm. The C4F [24] algorithm selects two times the standard deviation of the observation noise as the cutoff distance, the multi-source n-points [26] calculates the cutoff distance using an online learning algorithm, and the proposed algorithm selects 2% of the sorted distances matrix $d_{ij}$ (from small to large) as the cutoff distance. The multi-source n-points algorithm and the algorithm proposed in this paper can deal with unknown observation noise associated with the proposed clustering problem, whereas C4F can only deal with the case of known observation noise.*

**Remark** **2.** *An indispensable step in the existing multi-sensor data fusion clustering algorithm is to calculate the point-to-point distance, which is also the most time-consuming part of the algorithm. The runtime complexity/storage space requirements of the proposed algorithm and the multi-source n-points algorithm are $O(N^{2})$/$(N^{2}-N)/2$, O(NlogN)/O(N), respectively. Compared with the multi-source n-points algorithm, it can be seen that the proposed algorithm runs more slowly and requires more storage space.*

**Remark** **3.** *For the multi-source n-points algorithm, the selection of sensor s is very critical. If one target in sensor s is lost, this target will not be detected during the subsequent clustering process, while the proposed algorithm can well deal with the case of some targets avoiding detection. This is the advantage of the proposed algorithm, which is more obvious when the sensor detection probability is lower.*

## 4. Simulation Results

In this section, we compare the proposed algorithm with the *k*-means algorithm [8], multi-source n-points algorithm [26], and typical DBSCAN [6] algorithm to obtain the performance of the various algorithms. 

### 4.1. Given Cutoff Distance

**Figure 4:**
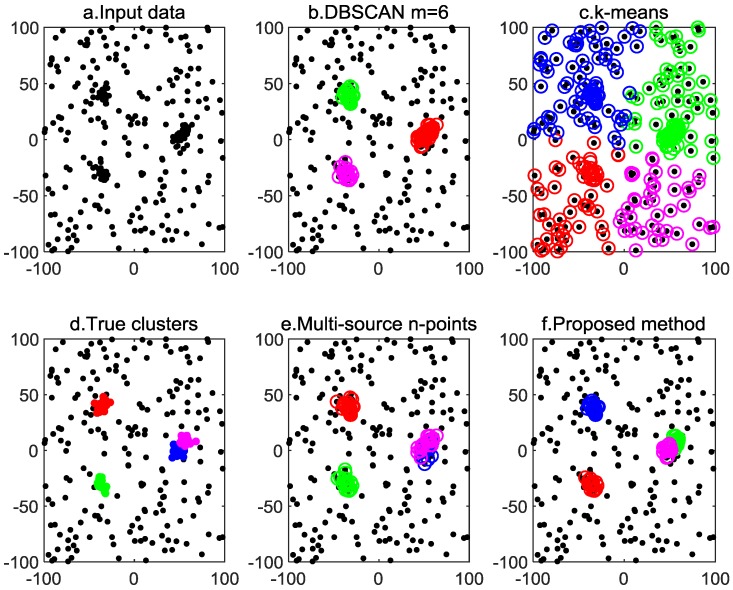
Outcomes of different clustering methods for data from 20 i.i.d sensors.

**Figure 5:**
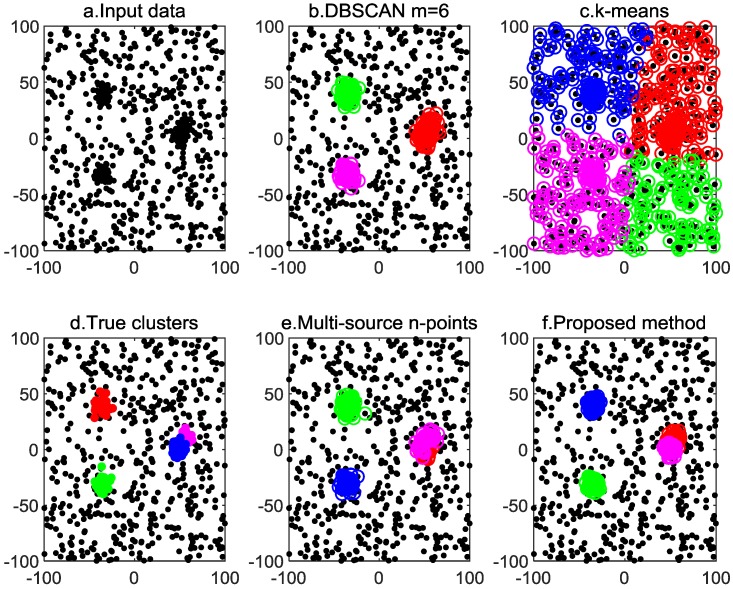
Outcomes of different clustering methods for data from 50 i.i.d.

The *k*-means clustering needs one parameter k (the number of clusters), and the DBSCAN algorithm needs two parameters ε (neighborhood radius) and m (minimum number of points). Both the multi-source n-points algorithm and the proposed algorithm need one parameter: the cutoff distance $d_{c}$. All the parameters used in the four algorithms are provided in Table 1. The number of sensors is set to n = {20, 50}, and the experimental results of n = {20, 50} are given in Figure 4 and Figure 5, respectively. 

In each Monte Carlo simulation, the color of the circles is assigned randomly, and circles of the same color represent the same cluster. The clustering results show that both the proposed method and the multi-source n-points algorithm can solve the MSDF clustering problem, but the proposed method algorithm has a smaller variance. The *k*-means algorithm is unable to deal with clutter, and the clustering result is incorrect. The DBSCAN algorithm can detect observations of targets, but the overlapping cluster clustering result is incorrect. 

Table 2 shows the average computing time of different algorithms for the 100 Monte Carlo simulations shown in Figure 4 and Figure 5. It shows that the proposed method is slower than the other three algorithms. To speed up the multi-sensor data fusion clustering algorithm, a target motion model can be employed to determine the potential cluster centroid.

### 4.2. Unknown Cutoff Distance

**Figure 6:**
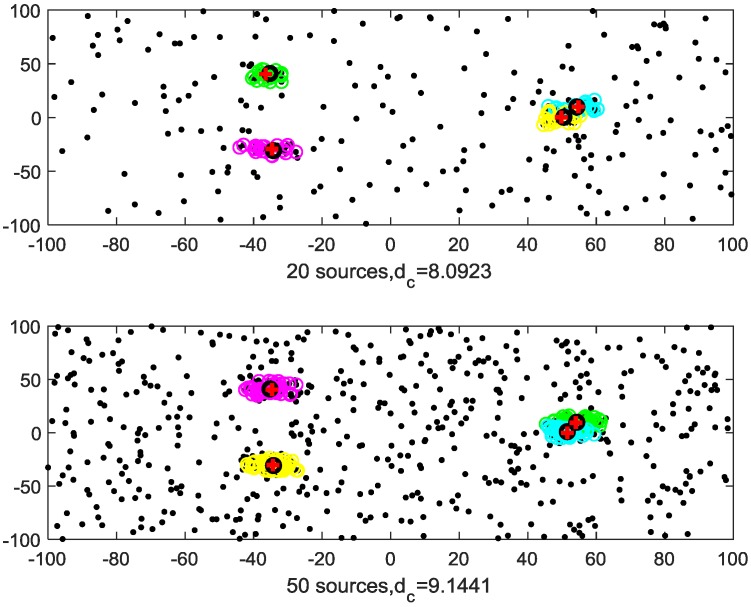
Clustering results with {20, 50} sensors (different color “O”) and the. Cluster centers, estimated using proposed method (black “O”) and multi-source n-points (red “+”).

Based on the same dataset as that given in Figure 2, we assume the cutoff distance $d_{c}$ is unknown and must be calculated from dataset using an algorithm, such as the DPC algorithm. The cutoff distances of the multi-source n-points and the proposed method are shown in Table 3. The clustering results of the proposed algorithm are given in Figure 6. Compared with the clustering results shown in Figure 4 and Figure 5, the clustering results shown in Figure 6 are also good. This demonstrates that the cutoff distance calculation used in the DPC algorithm is effective. 

### 4.3. Clustering-Based Model

**Figure 7:**
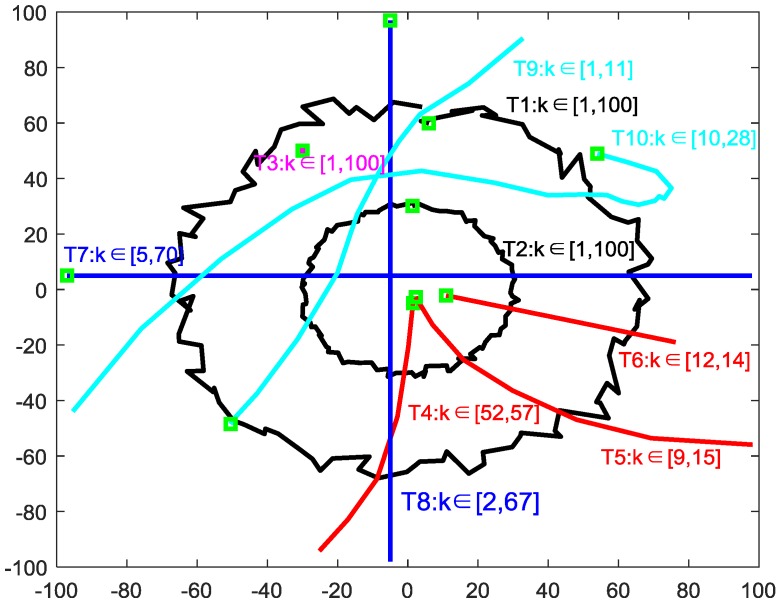
Trajectories of the targets with a fully unknown movement.

In this simulation, we compare our algorithm with the C4F and multi-source n-points algorithm for multiple target trajectories, provided in the excellent sample MATLAB code in [26]. Information on, for example, clutter and the target dynamic model, are unknown, and the only information that can be used is contained in the observations dataset (the data points in the two-dimensional Cartesian coordinate system) of multiple sensors. The surveillance area is [−100,100] × [−100,100] (m), and the start/end time and the initial position (green “□”) of each target are recorded near the target trajectory, as shown in Figure 7. The average clutter rate per scan is 10, and the observation noise obeys a zero-mean Gaussian distribution, with a variance of 4.

To test the clustering accuracy, we use the optimal sub-pattern assignment (OSPA) metric [29] to compare the proposed algorithm with the C4F and multi-source n-points algorithms. We set the cutoff parameter c = 100 and the ordered parameter p = 2. 

**Figure 8:**
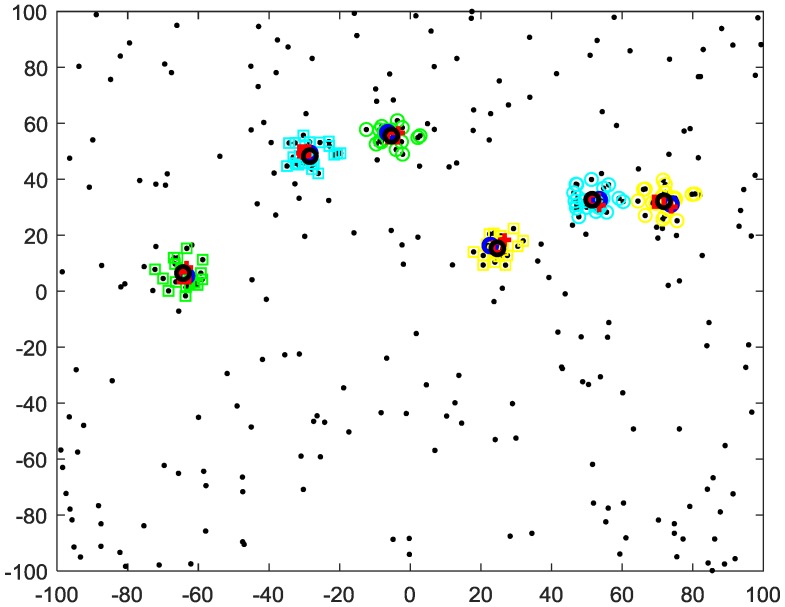
Clustering results of 20 sensors and different clusters (differently colored “o” and “□”), true target positions (red “□”), cluster centers of MS n-points (red “+”), C4F (blue “o”) and proposed method (black “o”).

**Figure 9:**
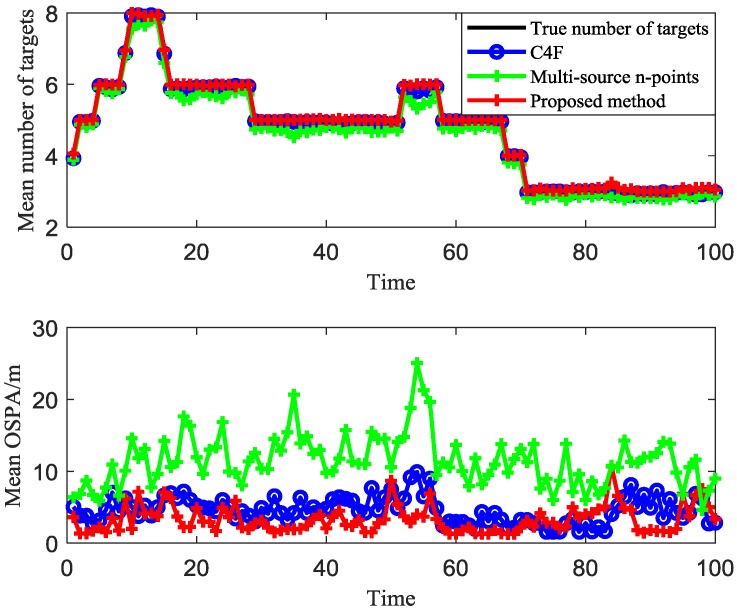
Mean estimated number of targets and mean OSPA of different algorithms over 20 MC trials.

First, we use 20 sensors. The clustering results of different algorithms for t = 16 are given in Figure 8. The average clustering target numbers and the average OSPA versus time over 100 Monte Carlo trails of different algorithms are given in Figure 9. The average OSPA of the proposed method is 3.7979, which is better than that of the C4F (5.9163) and the multi-source n-points (12.24).

**Figure 10:**
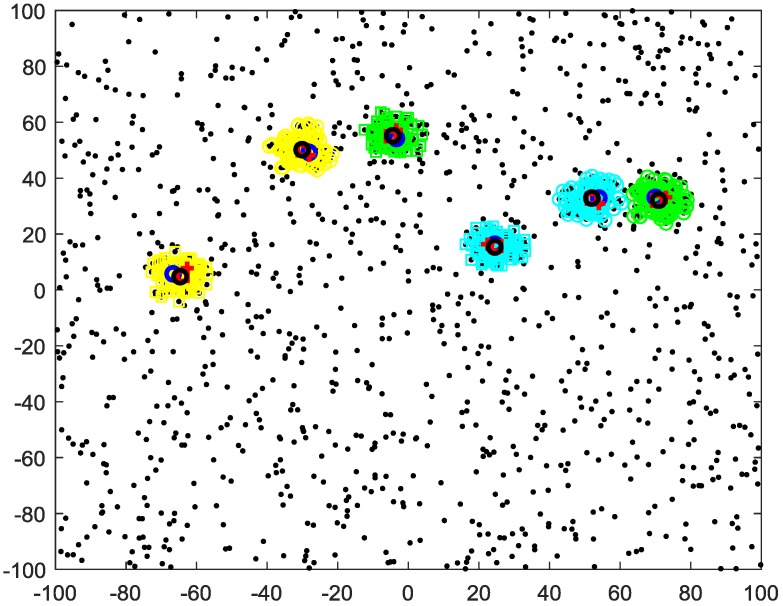
Clustering results of 100 sensors and different clusters (differently colored “o” and “□”), true target positions (red “□”), cluster centers of MS n-points (red “+”), C4F (blue “o”), and proposed method (black “o”).

**Figure 11:**
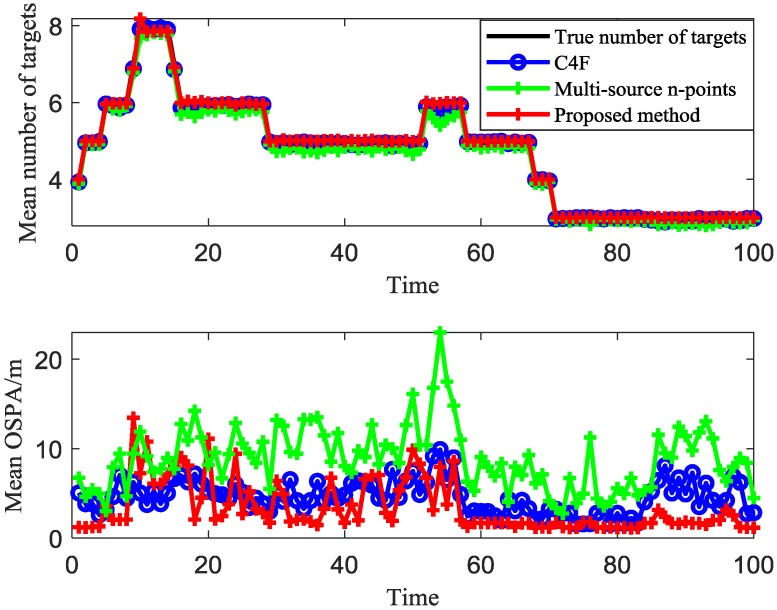
Mean estimated number of targets and mean OSPA of different algorithms over 100 MC trials.

Figure 10 shows the clustering results of different algorithms with 100 sensors for t = 16. The average clustering target number and the average OSPA comparison of different algorithms over 100 Monte Carlo trails are given in Figure 11. The average OSPA of the proposed method is 1.6717, which is better than that of the C4F (5.5241) and multi-source n-points (7.8788) algorithms. Compared with Figure 8, the clustering accuracy of the two algorithms increases with the increase of the sensor number.

**Figure 12:**
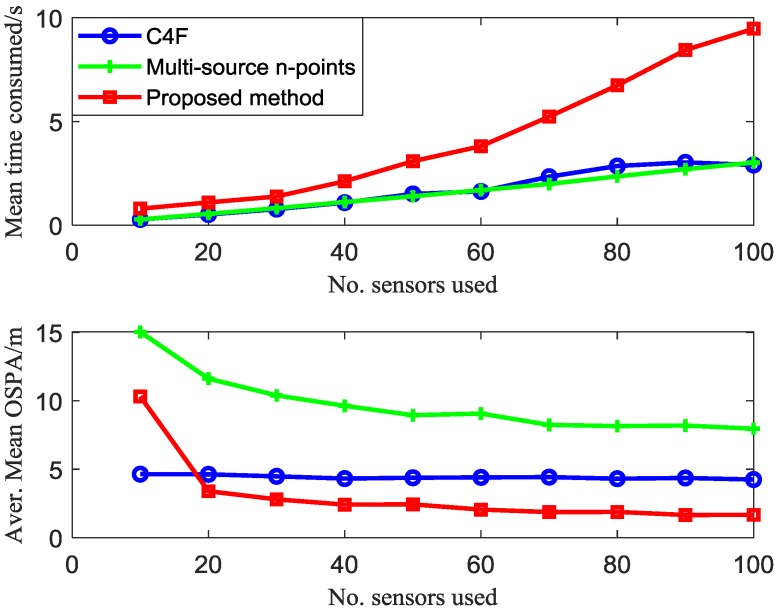
Mean OSPA and computing time of 100 steps × 100 MC runs, with different numbers of sensors.

Figure 12 gives the average time-consuming and average OSPA comparison of different algorithms versus different numbers of sensors over 100 Monte Carlo trials. It can be seen that the proposed method outperforms the C4F and multi-source n-points algorithm in the average OSPA. The clustering accuracy with 20 sensors using the proposed algorithm exceeds the clustering result with 100 sensors using the C4F and the multi-source n-points algorithms. As for the computing speed, the proposed method is slower than the C4F and multi-source n-points algorithms.

## 5. Conclusions

We propose a robust multi-sensor clustering algorithm to solve the MSDF problem. The MSDF problem corresponds to the clustering dataset of the observations (containing a large amount of noise) of multiple sensors, forming *k* clusters, and each cluster must satisfy the CL constraint. Unlike other model-based multi-sensor data fusion algorithms, no prior information, like the noise and motion model of a target, is needed in the proposed algorithm. Compared with the existing multi-sensor data fusion clustering algorithm, the proposed algorithm is more robust, and the lower the detection probability of the sensors, the better the performance of the proposed algorithm.

## Figures and Tables

**Table 1:** Parameters used in the four algorithms.

Algorithms	*k*-Means	DBSCAN	Multi-Source n-Points	Proposed Method
Parameters	k=4	ε=8/m=6	$d_{c}=8$	$d_{c}=8$

**Table 2:** Computing time of different clustering methods (s).

Algorithms	*k*-Means	DBSCAN m = 6	Multi-Source n-Points	Proposed Method
Figure 4	0.0059	0.0021	0.0051	**0.0078**
Figure 5	0.0087	0.0145	0.0198	**0.0346**

**Table 3:** Cutoff distance of different clustering methods (m).

Algorithms	Multi-Source n-Points	Proposed Method
20 sensors	6.7815	**8.0932**
50 sensors	5.9779	**9.1440**
